# Antioxidant and Anti-Inflammatory Activities of Six Flavonoids from *Smilax glabra* Roxb

**DOI:** 10.3390/molecules25225295

**Published:** 2020-11-13

**Authors:** Xinyu Zhao, Ruyi Chen, Yueyue Shi, Xiaoxi Zhang, Chongmei Tian, Daozong Xia

**Affiliations:** College of Pharmaceutical Sciences, Zhejiang Chinese Medical University, Hangzhou 310053, China; 201811113911453@zcmu.edu.cn (X.Z.); 201911113911468@zcmu.edu.cn (R.C.); 201911113911469@zcmu.edu.cn (Y.S.); 20201059@zcmu.edu.cn (X.Z.); m18042307982@163.com (C.T.)

**Keywords:** flavonoids, *Smilax glabra*, preparative HPLC, antioxidant, anti-inflammatory

## Abstract

This study aimed to isolate, prepare and identify the main flavonoids from a standardized *Smilax glabra* flavonoids extract (SGF) using preparative HPLC, MS, ^1^H NMR and ^13^C NMR, determine the contents of these flavonoids using UPLC, then compare their pharmacological activities in vitro. We obtained six flavonoids from SGF: astilbin (18.10%), neoastilbin (11.04%), isoastilbin (5.03%), neoisoastilbin (4.09%), engeletin (2.58%) and (−)-epicatechin (1.77%). The antioxidant activity of six flavonoids were evaluated by determining the 2,2-diphenyl-1-picrylhydrazyl (DPPH) radical and 2,2′-Azinobis (3-ethylbenzothiazoline-6-sulphonic acid) diammonium salt (ABTS^+^) radical scavenging activity and ferric reducing antioxidant power (FRAP). In addition, the anti-inflammatory activity of six flavonoids were evaluated by determining the production of cytokines (IL-1β, IL-6), nitric oxide (NO) using enzyme linked immunosorbent assay and the NF-κB p65 expression using Western blotting in lipopolysaccharide (LPS)-stimulated RAW264.7 cells. The results showed that (−)-epicatechin, astilbin, neoastilbin, isoastilbin and neoisoastilbin had strong antioxidant activities, not only in DPPH and ABTS^+^ radicals scavenging capacities, but in FRAP system. Furthermore, all the six flavonoids could significantly inhibit the secretion of IL-1β, IL-6, NO (*p* < 0.01) and the protein expression of NF-κB p-p65 (*p* < 0.01) in LPS-stimulated RAW264.7 cells. This study preliminarily verified the antioxidant and anti-inflammatory activities of six flavonoids in *S. glabra*.

## 1. Introduction

*Smilax glabra* Roxb. is a dried rhizome of Liliaceae plant. Hundreds of years ago, it had been used as food and folk medicine in some Asian countries, such as China, Japan, Korea and Vietnam. *S. glabra* was widely used in traditional Chinese medicine (TCM) for the treatment of syphilis, hypertonia, nephritis, heavy metal poisoning and other diseases in China [1,2]. In addition, it could be added to medicated diet as a functional ingredient. Modern pharmacological studies have shown that *S. glabra* has a variety of biological activities, such as antioxidant, anti-inflammatory, antiviral, antibacterial, hypouricemic, anti-gout, hepatoprotection and cardiovascular protection [3,4,5,6].

Flavonoids are the main active components in some medicinal plants and foods, which have various pharmacological functions [7,8]. *S. glabra* was rich in flavonoids. In previous investigations, several flavonoids compounds such as astilbin (2*R*,3*R*-taxifolin 3-*O*-α-l-rhamnopyranoside) and taxifolin have been isolated and identified from *S. glabra* [9,10]. Moreover, astilbin has four stereoisomers (astilbin, isoastilbin, neoisoastilbin, neoastilbin), which usually present simultaneously in plants [11]. Among them, astilbin was thought to be the main bioactive compound.

Free radicals are highly reactive intermediates with unpaired electrons. Free radical-mediated oxidative stress was involved in the pathogenesis of a variety of diseases, including cancer, inflammation and neurodegenerative disorders [12]. Some flavonoids (e.g. kaempferol, quercetin, quercetin 7-rhamnoside) as antioxidants could scavenge radicle species 2,2-diphenyl-1-picrylhydrazyl radical (DPPH radical) or 2,2′-Azinobis (3-ethylbenzothiazoline-6-sulphonic acid) diammonium salt (ABTS^+^ radical) in vitro [13,14].

Inflammation is an important contributor to the pathology of various acute and chronic diseases [15,16]. Lipopolysaccharide (LPS)-induced inflammation model in vitro could promote the release of inflammatory cytokines (IL-1β, IL-6, etc.) and the activation of nuclear factor-kappa B (NF-κB) pathway [17]. Some flavonoids (e.g., rutin, quercetin, morin) could inhibit the secretion of IL-1β, IL-6 and nitric oxide (NO), or block the phosphorylation of NF-κB p65 in RAW 264.7 cells [18,19].

According to TCM theory, the treatment of diseases by traditional Chinese materia medica based on the interaction of multiple components. Therefore, in our previous studies, a standardized *S. glabra* flavonoids extract (SGF) was prepared from the rhizomes of *S. glabra* [20], the flavonoids content was ~687 mg rutin equivalents/g dry extract [21]. In in vivo experiments, SGF has strong antioxidant, hepatoprotective, hypouricemic and nephroprotective effects [4,20], which has laid a foundation for this study.

In order to clarify the pharmacodynamic material basis and the potential pharmacological mechanisms of SGF, it is necessary to perform further isolation, preparation and identification of the main active compounds in SGF. Furthermore, as mentioned above, a wide variety of diseases are associated with oxidative stress caused by free radicals, as well as inflammation. Thus, this study also evaluated the antioxidant and anti-inflammatory activities of the main active compounds (six flavonoids) in SGF.

## 2. Results

### 2.1. Isolation of Six Flavonoids in SGF by Preparative High-performance Liquid Chromatography (PHPLC)

**Figure 1:**
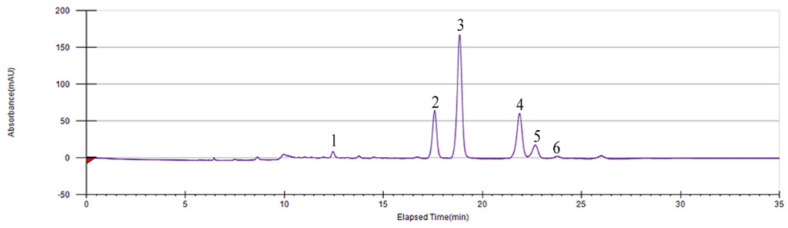
Isolation and preparation of the six main compounds in *Smilax glabra* flavonoids extract (SGF) by preparative high-performance liquid chromatography (PHPLC).

After optimized the isolation conditions of the six main compounds in SGF by ultra-high-pressure liquid chromatography (UPLC), the PHPLC method was used to isolate and purify these six compounds (Figure 1).

The six flavonoids in SGF were identified by the spectral data of Q-TOF MS, ^1^H-NMR and ^13^C-NMR (Supplementary Materials Figures S1–S4). The detailed data were as follows.

**Figure 2:**
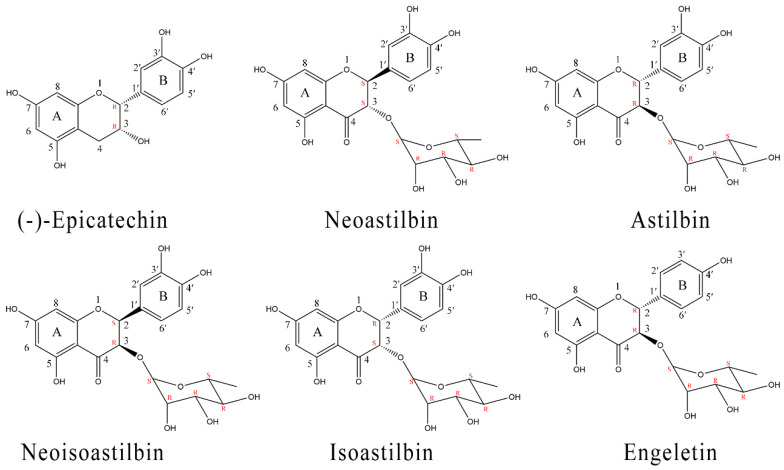
Chemical structures of six flavonoids in *Smilax glabra* flavonoids extract (SGF).

Compound **1** showed molecular ion [M − H]^−^ with *m*/*z* 289.0709, and the identified fragments were *m*/*z* 245.0807 [M − H − CO_2_]^−^. ^1^H NMR (DMSO-*d*_6_, 600 MHz, ppm), 2.46 (1H, dd, *J* = 3.6 Hz and 5.4 Hz, H-4), 2.67 (1H, dd, *J* = 4.8 Hz and 4.8 Hz, H-4), 4.00 (1H, ddd, H-3), 4.70 (1H, d, H-2), 4.73 (1H, s, 3-OH), 5.71 (1H, d, *J* = 2.4 Hz, H-6), 5.89 (1H, d, *J* = 2.4 Hz, H-8), 6.65 (2H, t, H-5′ and H-6′), 6.89 (1H, s, H-2′), 8.71 (1H, s, 3′-OH), 8.80 (1H, s, 4′-OH), 8.89 (1H, s, 5-OH), 9.10 (1H, s, 7-OH); ^13^C NMR (CD_3_OD, 150 MHz, ppm), 27.86 (C-4), 66.09 (C-3), 78.49 (C-2), 94.50 (C-8), 95.01 (C-6), 98.69 (C-10), 113.93 (C-2′), 114.50 (C-5′), 118.01 (C-6′), 130.90 (C-1′), 144.39 (C-3′), 144.55 (C-4′), 155.98 (C-5), 156.28 (C-7), 156.61 (C-9). MS, ^1^H and ^13^C NMR spectral data of the isolated compound agree well with the data reported [22]. From these data, it was identified as (−)-epicatechin ((−)-*cis*-3,3′,4′,5,7-pentahydroxyflavane)), the molecular formula was C_15_H_14_O_6_ (Figure 2).

Compound **2** showed molecular ion [M − H]^−^ with *m*/*z* 449.1076 and the identified fragments were *m*/*z* 304.0538 [M − H − Rha]^−^ and. *m/z* 285.0400 [M − H − Rha − OH]^−^. ^1^H NMR (DMSO-*d*_6_, 600 MHz, ppm), 0.79 (3H, d, *J* = 6 Hz, H-6″), 2.26 (1H, m, H-5″), 3.03 (1H, m, H-4″), 3.15 (2H, dd, *J* = 9.6, 3 Hz, H-3″ and , H-2″), 3.76 (1H, s), 4.45 (1H, d, *J* = 4.8Hz), 4.70 (1H, d, *J* = 6 Hz), 4.75 (1H, d, *J* = 11.2 Hz, H-3), 4.93 (1H, d, *J* = 4.8 Hz), 5.10 (1H, d, *J* = 11.2 Hz, H-2), 5.85 (1H, d, *J* = 2.4 Hz, H-6), 5.90 (1H, d, *J* = 2.4 Hz, H-8), 6.70 (2H, s, H-5′ and H-6′), 6.90 (1H, s, H-2′), 8.98 (1H, s, 3′-OH), 9.01 (1H, s, 4′-OH), 10.90 (1H, s, 7-OH), 11.72 (1H, s, 5-OH); ^13^C NMR (CD_3_OD, 150 MHz, ppm), 16.49 (C-6″), 68.90 (C-5″), 70.53 (C-2″), 72.04 (C-3″), 75.51 (C-4″), 82.28 (C-3), 94.90 (C-2), 96.06 (C-8), 100.65 (C-6), 101.44 (C-10), 114.09 (C-1″), 114.88 (C-5′), 119.57 (C-2′), 128.58 (C-6′), 145.23 (C-1′), 146.05 (C-3′), 162.91 (C-4′), 164.10 (C-9), 167.43 (C-5), 167.48 (C-7), 196.23 (C-4). MS, ^1^H and ^13^C NMR spectral data of the isolated compound agree well with the data reported [23]. From these data, it was identified as neoastilbin (2*S*,3*S*-taxifolin 3-*O*-α-l-rhamnopyranoside), the molecular formula was C_21_H_22_O_11_ (Figure 2).

Compound **3** showed molecular ion [M − H]^−^ with *m*/*z* 449.1084, and the identified fragments were *m*/*z* 304.0531 [M − H − Rha]^−^ and *m*/*z* 285.0394 [M − H − Rha − OH]^−^. ^1^H NMR (DMSO-*d*_6_, 600 MHz, ppm), 1.04 (3H, d, *J* = 6 Hz, H-6″), 3.12 (1H, d, *J* = 5.4 Hz, H-4″), 3.37 (1H, m, H-2″), 3.38 (1H, m, H-3″), 3.90 (1H, dd, *J* = 9.6, 3 Hz, H-5″), 4.03 (1H, s, H-1″), 4.51 (1H, d, *J* = 4.8 Hz), 4.52 (1H, d, *J* = 6 Hz), 4.65 (1H, d, *J* = 11.2 Hz, H-3), 4.71 (1H, d, *J* = 4.8 Hz), 5.24 (1H, d, *J* = 11.2 Hz, H-2), 5.88 (1H, d, *J* = 2.4 Hz, H-6), 5.90 (1H, d, *J* = 2.4 Hz, H-8), 6.73 (2H, s, H-5′ and H-6′), 6.88 (1H, s, H-2′), 9.54 (1H, s, 3′-OH), 9.97 (1H, s, 4′-OH), 10.88 (1H, s, 7-OH), 11.80 (1H, s, 5-OH); ^13^C NMR (CD_3_OD, 150 MHz, ppm), 16.45 (C-6″), 69.12 (C-5″), 70.38 (C-2″), 70.77 (C-3″), 72.41 (C-4″), 77.18 (C-3), 82.56 (C-2), 94.86 (C-8), 95.97 (C-6), 100.75 (C-1″), 101.10 (C-10), 114.09 (C-5′), 114.93 (C-2′), 119.09 (C-6′), 127.79 (C-1′), 145.15 (C-3′), 145.98 (C-4′), 162.71 (C-9), 164.12 (C-5), 167.19 (C-7), 194.60 (C-4). MS, ^1^H and ^13^C NMR spectral data of the isolated compound agree well with the data reported [24]. From these data, it was identified as astilbin (2*R*,3*R*-taxifolin 3-*O*-α-l-rhamnopyranoside), the molecular formula was C_21_H_22_O_11_ (Figure 2).

Compound **4** showed molecular ion [M − H]^−^ with *m*/*z* 449.1083 and the identified fragments were *m*/*z* 304.0536 [M − H − Rha]^−^ and *m*/*z* 285.0400 [M − H − Rha − OH]^−^. ^1^H NMR (DMSO-*d*_6_, 600 MHz, ppm), 1.00 (3H, d, *J* = 6 Hz, H-6″), 2.73 (1H, s, H-5″), 2.89 (1H, s, H-4″), 3.09 (1H, d, *J* = 5.4 Hz, H-3″), 3.46 (1H, s, H-2″), 4.10 (2H, q, *J* = 2.4 Hz), 4.50 (1H, d, *J* = 4.4 Hz), 4.59 (1H, d, *J* = 4.8 Hz, H-3), 4.69 (1H, d, *J* = 5.4 Hz), 5.47 (1H, d, *J* = 4.8 Hz, H-2), 5.92 (1H, s, H-6), 5.94 (1H, d, *J* = 2.4 Hz, H-8), 6.74 (2H, m, H-5′ and H-6′), 6.91 (1H, d, *J* = 1.8 Hz, H-2′), 8.99 (1H, s, 3′-OH), 9.00 (1H, s, 4′-OH), 10.91 (1H, s, 7-OH), 11.81 (1H, s, 5-OH); ^13^C NMR (CD_3_OD, 150 MHz, ppm), 16.11 (C-6″), 69.17 (C-5″), 70.18 (C-2″), 70.66 (C-3″), 72.34 (C-4″), 76.96 (C-3), 80.87 (C-2), 94.63 (C-8), 95,84 (C-6), 100.76 (C-1″), 101.18 (C-10), 113.87 (C-5′), 114.76 (C-2′), 118.04 (C-6′), 127.13 (C-1′), 144.91 (C-3′), 145.33 (C-4′), 163.06 (C-9), 164.62 (C-5), 167.36 (C-7), 192.47 (C-4). MS, ^1^H and ^13^C NMR spectral data of the isolated compound agree well with the data reported [25]. From these data, it was identified as neoisoastilbin (2*S*,3*R*-taxifolin 3-*O*-α-l-rhamnopyranoside), the molecular formula was C_21_H_22_O_11_ (Figure 2).

Compound **5** showed molecular ion [M − H]^−^ with *m*/*z* 449.1082, and the identified fragments were *m*/*z* 433.1134 [M − H − OH]^−^, *m*/*z* 304.0530 [M − H − Rha]^−^ and 285.0397 [M − H − Rh − OH]^−^. ^1^H NMR (DMSO-*d*_6_, 600 MHz, ppm), 0.84 (3H, d, *J* = 6 Hz, H-6″), 2.45 (1H, m, H-5″), 3.05 (1H, m, H-4″), 3.19 (1H, dd, *J* = 3, 3 Hz, H-3″), 3.46 (1H, s, H-2″), 4.21 (1H, d, *J* = 2.4Hz, H-3), 4.47 (2H, s, H-1″), 4.77 (1H, d, *J* = 18.0 Hz), 4.79 (1H, s), 5.55 (1H, d, *J* = 2.4 Hz, H-2), 5.92 (1H, d, *J* = 1.8 Hz, H-6), 5.95 (1H, d, *J* = 2.4 Hz, H-8), 6.72 (2H, m, H-5′ and H-6′), 6.84 (1H, s, H-2′), 8.89 (1H, s, 3′-OH), 9.00 (1H, s, 4′-OH), 10.95 (1H, s, 7-OH), 11.76 (1H, s, 5-OH); ^13^C NMR (CD_3_OD, 150 MHz, ppm), 16.37 (C-6″), 68.99 (C-5″), 70.60 (C-2″), 70.64 (C-3″), 71.94 (C-4″), 74.15 (C-3), 80.67 (C-2), 94.82 (C-8), 95.95 (C-6), 98.77 (C-1″), 100.39 (C-10), 113.83 (C-5′), 114.93 (C-2′), 117.97 (C-6′), 127.27 (C-1′), 144.96 (C-3′), 145.27 (C-4′), 163.09 (C-9), 164.75 (C-5), 167.36 (C-7), 192.94 (C-4). MS, ^1^H and ^13^C NMR spectral data of the isolated compound agree well with the data reported [26]. From these data, it was identified as isoastilbin (2*R*,3*S*-taxifolin 3-*O*-α-l-rhamnopyranoside), the molecular formula was C_21_H_22_O_11_ (Figure 2).

Compound **6** showed molecular ion [M − H]^−^ with *m*/*z* 433.1135and the identified fragments were *m*/*z* 269.0446 [M − H − Rha]^−^ and *m*/*z* 243.8981 [M − H − Rha − CO]^−^. ^1^H NMR (DMSO-*d*_6_, 600 MHz, ppm), 1.04 (3H, d, *J* = 6 Hz, H-6″), 3.11 (1H, m, H-5″), 3.26 (1H, m, H-4″), 3.38 (1H, m, H-3″), 3.90 (1H, dd, *J* = 9.6, 6 Hz, H-2″), 3.96 (1H, s), 4.49 (1H, d, *J* = 4.8Hz), 4.51 (1H, d, *J* = 6 Hz), 4.71 (1H, d, *J* = 6 Hz), 4.75 (1H, d, *J* = 10.2 Hz, H-3), 5.29 (1H, d, *J* = 10.2 Hz, H-2),5.88 (1H, d, *J* = 2.4 Hz, H-8), 5.91 (1H, d, *J* = 2.4 Hz, H-6), 6.78 (2H, d, *J* = 8.4 Hz, H-3′ and H-5′), 7.32 (2H, d, *J* = 8.4 Hz, H-2′ and H-6′), 9.63 (1H, s, 4-OH)10.95 (1H, s, 7-OH), 11.82 (1H, s, 5-OH); ^13^C NMR (CD_3_OD, 150 MHz, ppm), 16.45 (C-6″), 69.14 (C-5″), 70.38 (C-2″), 70.78 (C-3″), 72.40 (C-4″), 77.31 (C-3), 82.47 (C-2), 94.88 (C-8), 96.01 (C-6), 100.84 (C-10), 101.14 (C-1″), 115.05 (C-3′), 115.05 (C-5′), 127.22 (C-1′), 128.65 (C-2′), 128.65 (C-6′), 158.06 (C-4′), 162.74 (C-9), 164.12 (C-5), 167.16 (C-7), 194.67 (C-4). MS, ^1^H and ^13^C NMR spectral data of the isolated compound agree well with the data reported [27]. From these data, it was identified as engeletin (dihydrokaempferol 3-rhamnoside), the molecular formula was C_21_H_22_O_10_ (Figure 2).

**Figure 3:**
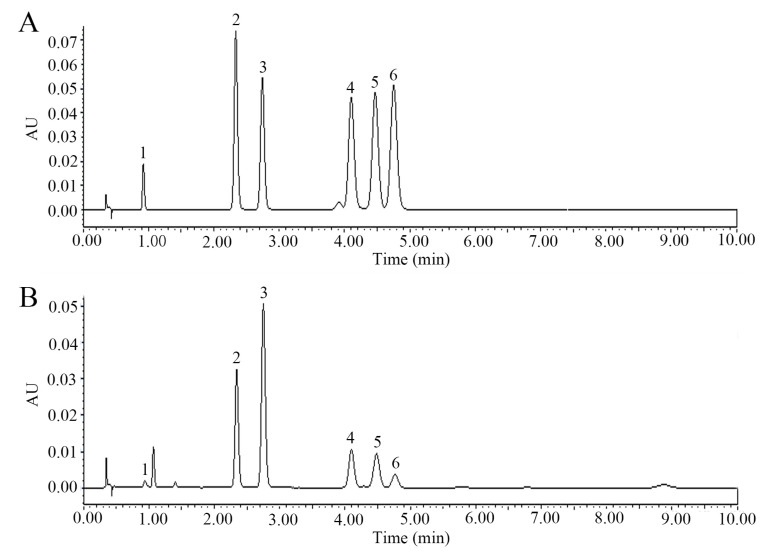
Quantitative analysis of six flavonoids in *Smilax glabra* flavonoids extract (SGF) by ultra-high-pressure liquid chromatography (UPLC). (**A**) Standards (purity ≥ 98%); (**B**) SGF sample. Compounds **1**, **2**, **3**, **4**, **5** and **6** represent for (−)-epicatechin, neoastilbin, astilbin, neoisoastilbin, isoastilbin and engeletin, respectively.

After quantitative analysis by UPLC, the percentage content of (−)-epicatechin, astilbin, neoastilbin, isoastilbin, neoisoastilbin, and engeletin in SGF was 1.77%, 18.10%, 11.04%, 5.03%, 4.09%, and 2.58%, respectively (Figure 3). Of these six flavonoids prepared from SGF, all the purities were more than 95%.

### 2.2. Antioxidant Activity of Six Flavonoids in SGF

**Figure 4:**
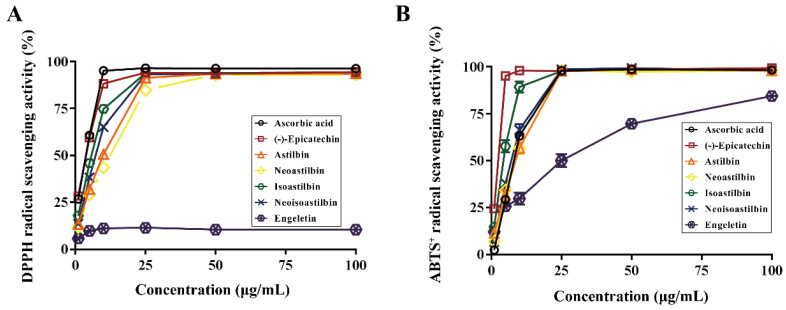
2,2-Diphenyl-1-picrylhydrazyl (DPPH) radical scavenging activity (**A**) and 2,2′-Azinobis (3-ethylbenzothiazoline-6-sulphonic acid) diammonium salt (ABTS^+^) radical scavenging activity (**B**) of six flavonoids in *Smilax glabra* flavonoids extract (SGF). Data are expressed as means ± SD (*n* = 3).

To evaluate the antioxidant activity of six flavonoids in SGF, we examined their DPPH radical and ABTS^+^ radical scavenging capacity, as well as the total reducing power. As shown in Figure 4 and Table 1, (−)-epicatechin, astilbin, neoastilbin, isoastilbin and neoisoastilbin showed strong DPPH radical scavenging activity with IC_50_ values 1.86 ± 0.22, 7.34 ± 0.22, 9.14 ± 0.23, 4.01 ± 0.18, 5.48 ± 0.22 μg/mL, respectively. However, engeletin had no obvious antioxidant activity in DPPH radical scavenging system. (−)-Epicatechin, astilbin, neoastilbin, isoastilbin, neoisoastilbin and engeletin showed strong ABTS^+^ radical scavenging activity with IC_50_ values 1.51 ± 0.13, 6.48 ± 1.13, 6.84 ± 0.55, 3.11 ± 0.90, 1.41 ± 0.55, 18.13 ± 1.72 μg/mL, respectively. Among them, (−)-epicatechin had the highest antioxidant activity (the smallest IC_50_) both in DPPH radical and in ABTS^+^ radical scavenging system; then followed by isoastilbin and neoisoastilbin. In order to evaluate the total reducing power of six flavonoids in SGF, we measured the ferric reducing antioxidant power (FRAP) using FeSO_4_ as the control. The results were consistent with the radicals scavenging experiments. As shown in Table 1, (−)-epicatechin, astilbin, neoastilbin, isoastilbin and neoisoastilbin in 50 μg/mL showed strong reducing power with FRAP values 499.33 ± 12.47, 148.22 ± 15.95, 223.78 ± 25.87, 400.44 ± 23.15, 421.56 ± 4.16 µM FeSO_4_ equivalent amount, respectively. However, engeletin had no obvious reducing power in this system. (−)-Epicatechin had the strongest reducing power (the highest FRAP value), then followed by isoastilbin and neoisoastilbin.

### 2.3. Anti-Inflammatory Activity of Six Flavonoids in SGF

**Figure 5:**
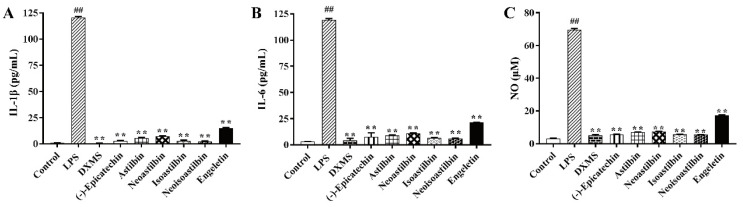
Effects of six flavonoids in *Smilax glabra* flavonoids extract (SGF) on the expression of inflammatory cytokines in RAW 264.7 cells. DXMS (dexamethasone) as positive control. RAW 264.7 cells were stimulated by lipopolysaccharide (LPS) (1 μg/mL) for 6 h, then treated with six flavonoids (100 μM) or DXMS (5 μM). The inflammatory cytokines levels of (**A**) IL-1β, (**B**) IL-6 and (**C**) NO in cell supernatant were detected by ELISA. Data are expressed as means ± SD (*n* = 3). *## p* < 0.01, compared with control group; ** p < 0.01, compared with LPS group.

**Figure 6:**
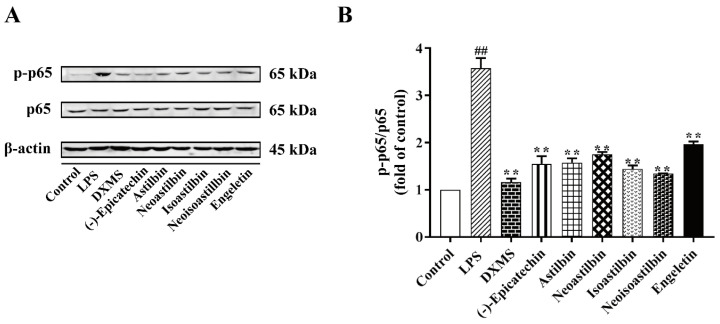
Effects of six flavonoids in *Smilax glabra* flavonoids extract (SGF) on the expression of NF-κB p-p65 in RAW 264.7 cells. DXMS (dexamethasone) as positive control. RAW 264.7 cells were stimulated by LPS (1 μg/mL) for 6 h, then treated with six flavonoids (100 μM) or DXMS (5 μM). (**A**) The protein expressions of NF-κB p-p65 and p65 detected by Western blotting. (**B**) The ratio of protein expression of NF-κB p-p65/p65. Data are expressed as means ± SD (*n* = 3). *## p* < 0.01, compared with control group; ** p < 0.01, compared with LPS group.

The anti-inflammatory activity of six flavonoids in SGF on LPS-stimulated RAW264.7 cells was investigated by measuring IL-1β, IL-6 and NO concentrations in cell culture supernatant. As shown in Figure 5, we found LPS increased the concentrations of IL-1β, IL-6 and NO significantly. (−)-Epicatechin, astilbin, neoastilbin, isoastilbin, neoisoastilbin and engeletin showed strong anti-inflammatory capacities compared to the LPS-stimulated RAW264.7 cells (*p* < 0.01). To further clarify the anti-inflammatory mechanism of six flavonoids, we determined the protein expression of NF-κB p-p65 in RAW 264.7 cells. As shown in Figure 6, LPS has induced NF-κB activation, which showed the protein expression of phosphorylated p65 (p-p65) in LPS-stimulated RAW264.7 cells increased significantly. (−)-Epicatechin, astilbin, neoastilbin, isoastilbin, neoisoastilbin and engeletin had obvious inhibitory effects on the protein expression of p-p65 (*p* < 0.01).

## 3. Discussion

Astilbin is a flavonoids compound. Previous studies have shown that astilbin was the main compound in *S. glabra*. However, the other stereoisomers of astilbin (isoastilbin, neoisoastilbin, neoastilbin) are naturally low in content [11]. Thus, this study aimed to isolate and prepare the flavonoids compounds from a standardized *S. glabra* flavonoids extract (SGF), instead of *S. glabra* raw material. We isolated and prepared six flavonoids using PHPLC from SGF: astilbin, neoastilbin, isoastilbin, neoisoastilbin, engeletin and (−)-epicatechin. The content of these six flavonoids was 18.10%, 11.04%, 5.03%, 4.09%, 2.58% and 1.77%, respectively. All the purities of these flavonoids were more than 95% which determined by UPLC. The results showed that astilbin was still the main chemical component in SGF. However, the percentage of the other three stereoisomers of astilbin in SGF was higher than in the raw material.

Some studies have shown that astilbin had good effects in the treatment of various diseases [6,10,27]. However, there were few studies on the pharmacological roles of the other chemical constituents in *S. glabra*. Excess radicals and oxidative stress caused a variety of diseases in the body. Previous investigations have indicated that reducing radicals could effectively prevent the occurrence of oxidative stress related diseases [28,29,30]. Thus, this study evaluated the antioxidant capacities of six flavonoids in SGF by determining the DPPH radical scavenging activity, the ABTS+ radical scavenging activity, as well as the ferric reducing antioxidant power (FRAP). The results showed that most of the isolated flavonoids had strong antioxidant activity. Among them, (−)-epicatechin, isoastilbin and neoisoastilbin showed stronger antioxidant activity than astilbin. The isomerism of natural compounds may affect their pharmacological activity to some extent [11]. The four stereoisomers of astilbin based on the C-2 and C-3 configuration, which is categorized in 4 stereoisomers: astilbin (2*R*,3*R*), neoisoastilbin (2*S*,3*R*), isoastilbin (2*R*,3*S*) and neoastilbin (2*S*,3*S*). The *trans*-stereoisomers of astilbin (neoisoastilbin, isoastilbin) showed higher antioxidant activities than *cis*-stereoisomers (astilbin, neoastilbin). Moreover, structure-activity studies of flavonoids indicated that the substitution patterns of B-ring could affect the antioxidant capacity [31]. The chemical structures of these flavonoids showed that the four stereoisomers of astilbin have 3′-OH and 4′-OH on B-ring, and the engeletin only has 4′-OH on B-ring. In addition, engeletin had no obvious antioxidant activity in DPPH radical system and showed low activities in ABTS^+^ radical scavenging and the FRAP evaluation, which indicated that 3′-OH on B-ring of flavonoids is more important to the antioxidant capacity.

In some diseases, oxidative stress and inflammation often occur simultaneously [32]. IL-1β, IL-6 and NO are the major mediators of inflammation in most inflammatory diseases. NF-κB was an important transcription factor with pro-inflammatory response. Upon activation, NF-κB moved rapidly into the nucleus and subsequently activated transcription of target genes, leading to the release of pro-inflammatory cytokines [33]. *S. glabra* was often used in the treatment of inflammatory diseases. Therefore, we also assessed the anti-inflammatory activity of six flavonoids in SGF by determining the cytokines (IL-1β, IL-6) and NO, and the expression of NF-κB p-p65/p65 in LPS-stimulated RAW 264.7 cells. The results showed that RAW 264.7 cells could release a variety of pro-inflammatory cytokines when stimulating by LPS, including IL-1β, IL-6 and NO, which were consistent with several literature reports [34,35]. Moreover, the production of IL-1β, IL-6, NO and phosphorylation of NF-κB (p-p65) in LPS-stimulated RAW264.7 cells were significantly inhibited by six flavonoids. The anti-inflammatory activities of astilbin and (−)-epicatechin were also consistent with several literature reports [27,36,37].

## 4. Materials and Methods 

### 4.1. Samples and Chemicals

The rhizome of *S. glabra* was obtained from a traditional Chinese medicine factory (Hangzhou, China), lot number 130101. It was identified by associate Prof. Chen Kongrong of Zhejiang Chinese Medical University. A voucher specimen was deposited at the Herbarium of College of Pharmaceutical Sciences at the Zhejiang Chinese Medical University (ZCPS7001). *S. glabra* flavonoids extract (SGF) was prepared as we described previously [4]. (−)-Epicatechin, neoastilbin, astilbin, neoisoastilbin, isoastilbin and engeletin were purchased from Sichuan Victory Biological Technology Co., Ltd. (Chengdu, China), with the purity more than 98%. Methanol and acetonitrile (chromatographic grade) were purchased from Merck (Darmstadt, Germany). Dexamethasone (DXMS), lipopolysaccharide (LPS), 2,2-diphenyl-1-picrylhydrazyl (DPPH), 2,2′-Azinobis (3-ethylbenzothiazoline-6-sulphonic acid) diammonium salt (ABTS) and TPTZ (2,4,6-tripyridyl-s-triazine) were purchased from Sigma-Aldrich (St. Louis, MO, USA). The RAW264.7 cell line (TIB-71) was purchased from American Type Culture Collection (Manassas, VA, USA). Iron chloride hexahydrate was purchased from Aladdin (Shanghai, China). Enzyme-linked immunosorbent assay (ELISA) kits for NO, IL-1β, IL-6 were purchased from MEIMIAN (Shanghai, China). NF-κB p-p65, NF-κB p65 and β-actin antibodies were purchased from Cell Signaling Technology (Boston, MA, USA).

### 4.2. Isolation of Six Flavonoids in SGF by Preparative High-Performance Liquid Chromatography (PHPLC)

In order to optimize the isolation conditions of the six flavonoids in SGF, ultra-high-pressure liquid chromatography (UPLC) with ACQUITY UPLC H-Class system (Waters, Milford, MA, USA) was performed. The SGF was dissolved with methanol and centrifuged to obtain the supernatant. Chromatographic conditions: Ultra High-performance Liquid Chromatography System (Waters, USA); ACQUITY UPLC BEH C_18_ column (2.1 × 50 mm, 1.7 µm); column temperature, 30 °C; and the mobile phase: CH_3_CN (A) and H_2_O with 0.3% formic acid (B). Gradient elution: 14.8% A, 0–6 min, 14.8–100% A, 6-6.01 min, 100% A, 6.01–10 min, 100–14.8% A, 10–15 min. The injected sample volume flow was 1μL. The temperature of the auto-sampler was maintained at RT, and the volume flow rate was 0.35 mL/min. Detection wavelength was 290 nm.

Then, isolation and preparation of six flavonoids in SGF was executed on Prep 150 LC system (Waters, Milford, MA, USA) consisted of a Waters 2545Q preparative pump-equipped with a Waters 2489 UV/Visible Detector-, a Waters Fraction Collector III-, Waters 2707 Automatic Sampler and a preparative column (Waters Sunfire Prep C_18_ OBDTM 250 × 19 mm, 5 μm). The PHPLC condition for six flavonoids was as follows:

CH_3_CN (A) and H_2_O with 0.3% formic acid (B). Gradient elution: 10% A, 100 mL/min, 0–5 min; 10–18% A, 10-35 mL/min, 5-5.01 min; 18% A, 35 mL/min, 5.01–20 min; 18–95% A, 35 mL/min, 20–20.1 min; 95% A, 35 mL/min, 20.01–25 min; 95–10% A, 35-10 mL/min, 25–25.01 min; 10% A, 10 mL/min, 25–35 min. The detection wavelength and injection volume were 290 nm and 500 μL, respectively.

The isolated samples were lyophilized for further identification of structure. In this experiment, the six flavonoids were identified by confrontation of the spectral characteristics (Waters UPLC Synapt G2-S mass spectrometer (Waters, Milford, MA, USA). MS conditions: electrospray ionization (ESI), negative ion mode, drying gas flow rate is 11 L/min, drying gas temperature is 300 °C, capillary voltage is 3500 V, the nebulizer pressure is 25 psig, the fragmentor voltage is 175 V, the skimmer voltage is 60 V. ^1^H-NMR and ^13^C-NMR, Bruker 600 MHz NMR spectrometer (Bruker, Rheinstetten, Germany)) with those depicted in a previous study. The quantitative analysis and purity of six flavonoids in SGF were determined by ACQUITY UPLC H-Class system mentioned above.

### 4.3. The Antioxidant Effect of the Six Flavonoids in SGF

#### 4.3.1. Sample Preparation

Six flavonoids isolated from SGF were dissolved in methanol to 1000 μg/mL and diluted to 100, 50, 25, 10, 5 and 1 μg/mL, respectively. Ascorbic acid was dissolved in deionized water to 1000 μg/mL and diluted to 100, 50, 25, 10, 5 and 1 μg/mL.

#### 4.3.2. DPPH Radical Scavenging Activity

DPPH radical has the maximal absorbance at 517 nm, the absorption value could be decreased by the antioxidant compounds. The capacity of six flavonoids in SGF to remove DPPH radical was determined according to a previously described procedure with slight modifications [38]. DPPH radical (20 mg) was accurately weighed and dissolved in absolute alcohol to 200 μM. Different concentrations of sample solutions (2 mL) were mixed with DPPH radical solution (2 mL). The mixture was incubated in the dark for 30 min at room temperature after vortex mixing, followed by measurement at 517 nm using UV-VIS 3600 spectrophotometer (Shimadzu, Tokyo, Japan), the absorbance is A_i_. Moreover, the absorbance of the mixture of different concentrations of sample solutions (2 mL) and absolute alcohol (2 mL) were determined as A_j_. The absorbance of the mixture of DPPH radical solution (2 mL) and methanol (2 mL) were determined as A_c_. Ascorbic acid standard was used for comparison.

DPPH radical scavenging activity (%) = [(A_i_ − A_j_)/A_c_] × 100

#### 4.3.3. ABTS^+^ Radical Scavenging Activity

ABTS^+^ radical has the maximal absorbance at 734 nm, the absorption value could be decreased by the antioxidant compounds. The radical scavenging activity of six flavonoids in SGF for ABTS^+^ radical was determined by a previously described procedure [38]. ABTS^+^ radicle was dissolved in deionized water to 7 mM, and the stock solution were generated by reacting ABTS^+^ radical stock solution with 2.45 mM potassium persulfate in the dark for 16 h at room temperature. The working solution was diluted in ethanol to an absorbance of 0.7 ± 0.02 at 734 nm. ABTS^+^ radical solution (3.6 mL) was mixed with different concentrations of sample solutions. Then, the mixture was incubated in the dark for 10 min at room temperature after vortex mixing, and the absorbance of the mixture was determined as A_i_. Moreover, the absorbance of the mixture of ABTS^+^ radical solution (3.6 mL) and methanol (0.4 mL) was determined as A_j_. Ascorbic acid standard was used for comparison.

ABTS^+^ radical scavenging activity (%) = [1 − A_i_/A_j_] × 100

#### 4.3.4. Ferric Reducing Antioxidant Power (FRAP) Assay

The FRAP value of six flavonoids in SGF was determined by a previously described procedure with minor modifications [39]. FRAP working solution: 300 mM acetate buffer, 10 mM TPTZ diluted in 40 mM HCl and 20 mM FeCl_3_·6H_2_O solution were mixed at 10:1:1.

FeSO_4_ standard curve: FeSO_4_ was dissolved in deionized water to 4000 μM and diluted to 1000, 800, 400, 200, 100 and 25 μM. Different concentrations of FeSO_4_ (0.1 mL) was mixed with FRAP working solution (3 mL) and deionized water (0.3 mL) at 37 °C water bath for 4 min, then determined the absorbance at 593 nm. The standard curve (R^2^ = 0.9993) was linear in the range 0–4000 µM.

FRAP values of samples: Take 0.1 mL 50 μg/mL samples, mixed with FRAP working solution (3 mL) and deionized water (0.3 mL) at 37 °C water bath for 4 min, then determined the absorbance at 593 nm. The total reducing power (FRAP values) was calculated according to the standard curve (µM FeSO_4_ equivalent amount).

### 4.4. The Anti-Inflammatory Effect of Compounds in TFSG on RAW 264.7 Cells

#### 4.4.1. RAW 264.7 Cells Culture and Treatment

RAW 264.7 cells were cultured in Dulbecco’s Modified Eagle Medium (DMEM) contained with 10% fetal bovine serum (FBS), 100 µg/mL streptomycin and 100 U/mL penicillin. The cell suspension (1 × 10^5^ cells/mL) was added to a 96-well plate (100 µL/well) at 37 °C, 5% CO_2_ for 24 h, then treated with 100 μM (−)-epicatechin, neoastilbin, astilbin, neoisoastilbin, isoastilbin or engeletin for 0.5 h, followed by stimulated with LPS (1 μg/mL) and incubated for another 6 h. Dexamethasone (DXMS, 5 μM) as positive control [40].

#### 4.4.2. Inflammatory Cytokines Determination by Enzyme Linked Immunosorbent Assay (ELISA)

The contents of IL-1β, IL-6 and NO were determined according to the manufacturer’s protocols [19,41]. In short, 50 µL of the cell supernatant was added to a new 96-well plate, then incubated at 37 °C for 0.5 h. After washing 5 times using washing buffer (each wash lasts 30 s), 50 μL of HRP-conjugate reagent was added to each well and incubate at 37 °C for 0.5 h. After washing 5 times, 50 μL of chromogen solution A and B were added to each well, then protected from light and incubated at 37 °C for 10 min. Finally, 50 μL of stop solution was added to each well. The absorbance was read at 450 nm within 15 min.

#### 4.4.3. NF-κB p-p65 Expression Assay by Western Blotting

RAW264.7 cells protein lysis was collected and the concentration of protein was measured after centrifugation at 12,000 rpm for 10 min at 4 °C. Then, the sample proteins (20 μg) were electrophoresed and separated in 10% polyacrylamide gel prior to being transferred onto PVDF membranes. After transferring the target proteins to PVDF membranes, the membranes were blocked with 5% BSA in TBST for 1 h, then incubated with anti-NF-κB p65 (dilution, 1:1000; CST), anti-NF-κB p-p65 (dilution, 1:1000; CST) and anti-actin (dilution, 1:5000; CST) at 4 °C for overnight [32]. Finally, the PVDF membranes were incubated with the anti-mouse or anti-rabbit IgG for 2 h at room temperature and detected by the Two-color Infrared Laser Imaging System (LI-COR Odyssey Clx).

### 4.5. Statistical Analysis

The results were analyzed by one-way analysis of variance (ANOVA), followed by a post hoc test using the SPSS 20.0 software (IBM Corp., Armonk, NY, USA). The data are the mean ± SD of three independent experiments. *p* < 0.05 was considered significant.

## 5. Conclusions

In summary, we identified six flavonoids from SGF: astilbin (18.10%), neoastilbin (11.04%), isoastilbin (5.03%), neoisoastilbin (4.09%), engeletin (2.58%) and (−)-epicatechin (1.77%). Moreover, we found that (−)-epicatechin, astilbin, neoastilbin, isoastilbin and neoisoastilbin had strong antioxidant activitiesboth in DPPH and ABTS^+^ radicals scavenging capacitiesand in FRAP system. All the six flavonoids could inhibit the secretion of IL-1β, IL-6, NO and protein expression of NF-κB p-p65 in LPS-stimulated RAW264.7 cells. This study laid a foundation for future research on the potential pharmacological mechanisms and new product development of these six flavonoids and *S. glabra*.

## Figures and Tables

**Table 1:** IC_50_ of DPPH and ABTS^+^ radicals scavenging activity and FRAP values of six flavonoids in *Smilax glabra* flavonoids extract (SGF).

	Ascorbic Acid	(−)-Epicatechin	Astilbin	Neoastilbin	Isoastilbin	Neoisoastilbin	Engeletin
IC_50_ of DPPH radical (μg/mL)	1.90 ± 0.14	1.86 ± 0.22	7.34 ± 0.22	9.14 ± 0.23	4.01 ± 0.18	5.48 ± 0.22	>100
IC_50_ of ABTS^+^ radical (μg/mL)	7.26 ± 0.18	1.51 ± 0.13	6.48 ± 1.13	6.84 ± 0.55	3.11 ± 0.90	1.41 ± 0.55	18.1 ± 1.7
FRAP values (µM FeSO_4_ equivalent amount)	713 ± 31	499 ± 12	148 ± 16	224 ± 26	400 ± 23	421 ± 4	16.0 ± 2.7

Data are expressed as means ± SD (*n* = 3).

## Supplementary Materials

The Supplementary Materials are available online. Figure S1: The mass spectra of six flavonoids in *Smilax glabra* flavonoids extract (SGF). Figure S2: The ^1^H NMR spectra of six flavonoids in *Smilax glabra* flavonoids extract (SGF). Figure S3: The ^13^C NMR spectra of six flavonoids in *Smilax glabra* flavonoids extract (SGF). Figure S4: The total ion current (TIC) of *Smilax glabra* flavonoids extract (SGF).

## References

[1] Hua S., Zhang Y., Liu J., Dong L., Huang J., Lin D., Fu X. (2018). Ethnomedicine, phytochemistry and pharmacology of *Smilax glabra*: An important traditional Chinese medicine. Am. J. Chin. Med..

[2] Bao Y., Li H., Li Q.Y., Li Y., Li F., Zhang C.F., Wang C.Z., Yuan C.S. (2018). Therapeutic effects of *Smilax glabra* and *Bolbostemma paniculatum* on rheumatoid arthritis using a rat paw edema model. Biomed. Pharmacother..

[3] Feng H., He Y., La L., Hou C., Song L., Yang Q., Wu F., Liu W., Hou L., Li Y. (2020). The flavonoid-enriched extract from the root of *Smilax china* L. inhibits inflammatory responses via the TLR-4-mediated signaling pathway. J. Ethnopharmacol..

[4] Xia D., Fan Y., Zhang P., Fu Y., Ju M., Zhang X. (2013). Protective effects of the flavonoid-rich fraction from rhizomes of *Smilax glabra* Roxb. on carbon tetrachloride-induced hepatotoxicity in rats. J. Membr. Biol..

[5] Lu C., Zhu W., Wang M., Xu X., Lu C. (2014). Antioxidant and anti-inflammatory activities of phenolic-enriched extracts of *Smilax glabra*. Alternat. Med..

[6] Huang L., Deng J., Chen G., Zhou M., Liang J., Yan B., Shu J., Liang Y., Huang H. (2019). The anti-hyperuricemic effect of four astilbin stereoisomers in *Smilax glabra* on hyperuricemic mice. J. Ethnopharmacol..

[7] Kerimi A., Williamson G. (2018). Differential impact of flavonoids on redox modulation, bioenergetics, and cell signaling in normal and tumor cells: A comprehensive review. Antioxid. Redox Signal..

[8] Chen W.D., Zhao Y.L., Sun W.J., He Y.J., Liu Y.P., Jin Q., Yang X.W., Luo X.D. (2020). “Kidney Tea” and its bioactive secondary metabolites for treatment of gout. J. Agric. Food Chem..

[9] Shu J., Li L., Zhou M., Yu J., Peng C., Shao F., Liu R., Zhu G., Huang H. (2018). Three new flavonoid glycosides from *Smilax glabra* and their anti-inflammatory activity. Nat. Prod. Res..

[10] Xu S., Shang M., Liu G., Xu F., Wang X., Shou C., Cai S. (2013). Chemical constituents from the rhizomes of *Smilax glabra* and their antimicrobial activity. Molecules.

[11] Zheng D., Zhang L., Zhang Q. (2018). Isomerization of astilbin and its application for preparation of the four stereoisomers from *Rhizoma Smilacis Glabrae*. J. Pharmaceut. Biomed..

[12] McCord J.M. (2000). The evolution of free radicals and oxidative stress. Am. J. Med..

[13] Piccolella S., Fiorentino A., Pacifico S., D'Abrosca B., Uzzo P., Monaco P. (2008). Antioxidant properties of Sour Cherries (*Prunus cerasus* L.): Role of colorless phytochemicals from the methanolic extract of ripe fruits. J. Agr. Food Chem..

[14] Huang Z.Q., Chen P., Su W.W., Wang Y.G., Wu H., Peng W., Li P.B. (2018). Antioxidant activity and hepatoprotective potential of quercetin 7-rhamnoside in vitro and in vivo. Molecules.

[15] Gupta S.C., Kunnumakkara A.B., Aggarwal S., Aggarwal B.B. (2018). Inflammation, a double-edge sword for cancer and other age-related diseases. Front. Immunol..

[16] Oikonomou E., Tousoulis D. (2018). Inflammation: A pathogenetic mechanism or a mediator, linking risk factors and cardiovascular disease?. Int. J. Cardiol..

[17] Rozenberg K., Wollman A., Ben-Shachar M., Argaev-Frenkel L., Rosenzweig T. (2019). Anti-inflammatory effects of *Sarcopoterium spinosum* extract. J. Ethnopharmacol..

[18] Chen Y.C., Shen S.C., Lee W.R., Hou W.C., Yang L.L., Lee T.J.F. (2001). Inhibition of nitric oxide synthase inhibitors and lipopolysaccharide induced inducible NOS and cyclooxygenase-2 gene expressions by rutin, quercetin, and quercetin pentaacetate in RAW 264.7 macrophages. J. Cell. Biochem..

[19] Dhanasekar C., Kalaiselvan S., Rasool M. (2015). Morin, a bioflavonoid suppresses monosodium urate crystal-induced inflammatory immune response in RAW 264.7 macrophages through the inhibition of inflammatory mediators, intracellular ROS levels and NF-kappaB activation. PLoS ONE.

[20] Wang S., Fang Y., Yu X., Guo L., Zhang X., Xia D. (2019). The flavonoid-rich fraction from rhizomes of *Smilax glabra* Roxb. ameliorates renal oxidative stress and inflammation in uric acid nephropathy rats through promoting uric acid excretion. Biomed. Pharmacother..

[21] Shi Y., Tian C., Yu X., Fang Y., Zhao X., Zhang X., Xia D. (2020). Protective effects of *Smilax glabra* Roxb. against lead-induced renal oxidative stress, inflammation and apoptosis in weaning rats and HEK-293 cells. Front. Pharmacol..

[22] Davis A., Cai Y., Davies A., Lewis J. (1996). ^1^H and ^13^C NMR assignments of some green tea polyphenols. Magn. Reson. Chem..

[23] Xu Y., Capistrano R., Dhooghe L., Foubert K., Lemière F., Maregesi S., Baldé A., Apers S., Pieters L. (2011). Herbal medicines and infectious diseases: Characterization by LC-SPE-NMR of some medicinal plant extracts used against malaria. Planta Med..

[24] Gu W., Li N., Leung E., Zhou H., Yao X., Liu L., Wu J. (2015). Rapid identification of new minor chemical constituents from *Smilacis glabrae* Rhizoma by combined use of UHPLC-Q-TOF-MS, preparative HPLC and UHPLC-SPE-NMR-MS techniques: Rapid identification of new minor constituents by LC-MS and LC-SPE-NMR. Phytochem. Anal..

[25] Guo W., Dong H., Wang D., Yang B., Wang X., Huang L. (2018). Separation of seven polyphenols from the rhizome of *Smilax glabra* by Offline Two Dimension Recycling HSCCC with extrusion mode. Molecules.

[26] Zhou X., Xu Q., Li J., Chen T. (2009). Structural revision of two flavanonol glycosides from *Smilax glabra*. Planta Med..

[27] Xin W., Huang H., Yu L., Shi H., Sheng Y., Wang T., Yu L. (2012). Three new flavanonol glycosides from leaves of *Engelhardtia roxburghiana*, and their anti-inflammation, antiproliferative and antioxidant properties. Food Chem..

[28] Kehrer J.P., Klotz L.O. (2015). Free radicals and related reactive species as mediators of tissue injury and disease: Implications for health. Crit. Rev. Toxicol..

[29] Lu C., Zhu Y., Hu M., Wang D., Xu X., Lu C., Zhu W. (2015). Optimization of astilbin extraction from the rhizome of *Smilax glabra*, and evaluation of its anti-inflammatory effect and probable underlying mechanism in lipopolysaccharide-induced RAW264.7 macrophages. Molecules.

[30] Barzegar A.O.M., Schiesser C.H., Taylor M.K. (2014). New reagents for detecting free radicals and oxidative stress. Org. Biomol. Chem..

[31] Arora A., Nair M.G., Strasburg G.M. (1998). Structure-activity relationships for antioxidant activities of a series of flavonoids in a liposomal system. Free Radic. Biol. Med..

[32] Poprac P., Jomova K., Simunkova M., Kollar V., Rhodes C.J., Valko M. (2017). Targeting free radicals in oxidative stress-related human diseases. Trends Pharmacol. Sci..

[33] Napetschnig J., Wu H. (2013). Molecular basis of NF-κB signaling. Annu. Rev. Biophys..

[34] Mitchell S., Vargas J., Hoffmann A. (2016). Signaling via the NF-κB system. Wiley Interdiscip. Rev. Syst. Biol. Med..

[35] Lee J., Li C., Surayot U., Yelithao K., Lee S., Park W., Tabarsa M., You S. (2018). Molecular structures, chemical properties and biological activities of polysaccharide from *Smilax glabra* rhizome. Int. J. Biol. Macromol..

[36] Wu H., Xie Y., Xu Y., Hu Z., Wan X., Huang H., Huang D. (2020). Protective effect of epicatechin on APAP-induced acute liver injury of mice through anti-inflammation and apoptosis inhibition. Nat. Prod. Res..

[37] Fraga C., Oteiza P., Galleano M. (2018). Plant bioactives and redox signaling: (-)-Epicatechin as a paradigm. Mol. Aspects Med..

[38] Shi J.Y., Gong J.Y., Liu J.E., Wu X.Q., Zhang Y. (2009). Antioxidant capacity of extract from edible flowers of *Prunus mume* in China and its active components. LWT Food Sci. Technol..

[39] Xia D.Z., Yu X.F., Zhu Z.Y., Zou Z.D. (2011). Antioxidant and antibacterial activity of six edible wild plants (*Sonchus spp*.) in China. Nat. Prod. Res..

[40] Gong G., Xie F., Zheng Y., Hu W., Qi B., He H., Dong T.T., Tsim K.W. (2020). The effect of methanol extract from *Saussurea involucrata* in the lipopolysaccharide-stimulated inflammation in cultured RAW 264.7 cells. J. Ethnopharmacol..

[41] Lee S.G., Brownmiller C.R., Lee S.O., Kang H.W. (2020). Anti-inflammatory and antioxidant effects of anthocyanins of *Trifolium pratense* (Red Clover) in lipopolysaccharide-stimulated RAW-267.4 macrophages. Nutrients.

