# Supplementary material for: Antioxidant and Anti-Inflammatory Activities of Six Flavonoids from Smilax glabra Roxb
Source: Molecules. 2020 Nov 13;25(22):5295. doi: 10.3390/molecules25225295 (PMC7697956; doi:10.3390/molecules25225295)
Supplement: Supplementary file 1 [file molecules-25-05295-s001.pdf]

Supplementary Materials: Antioxidant and anti-inflammatory activities of six flavonoids from Smilax glabra Roxb

Xinyu Zhao †, Ruyi Chen †, Yueyue Shi †, Xiaoxi Zhang, Chongmei Tian and Daozong Xia *

College of Pharmaceutical Sciences, Zhejiang Chinese Medical University, Hangzhou 310053, China; 201811113911453@zcmu.edu.cn (X.Z.); 201911113911468@zcmu.edu.cn (R.C.); 201911113911469@zcmu.edu.cn (Y.S.); 20201059@zcmu.edu.cn (X.Z.); m18042307982@163.com (C.T.)

* Correspondence: xdz_zjtcm@hotmail.com or xiadaozong@zcmu.edu.cn; Tel./Fax: +86-571-61768535

† These authors contributed equally to this work.

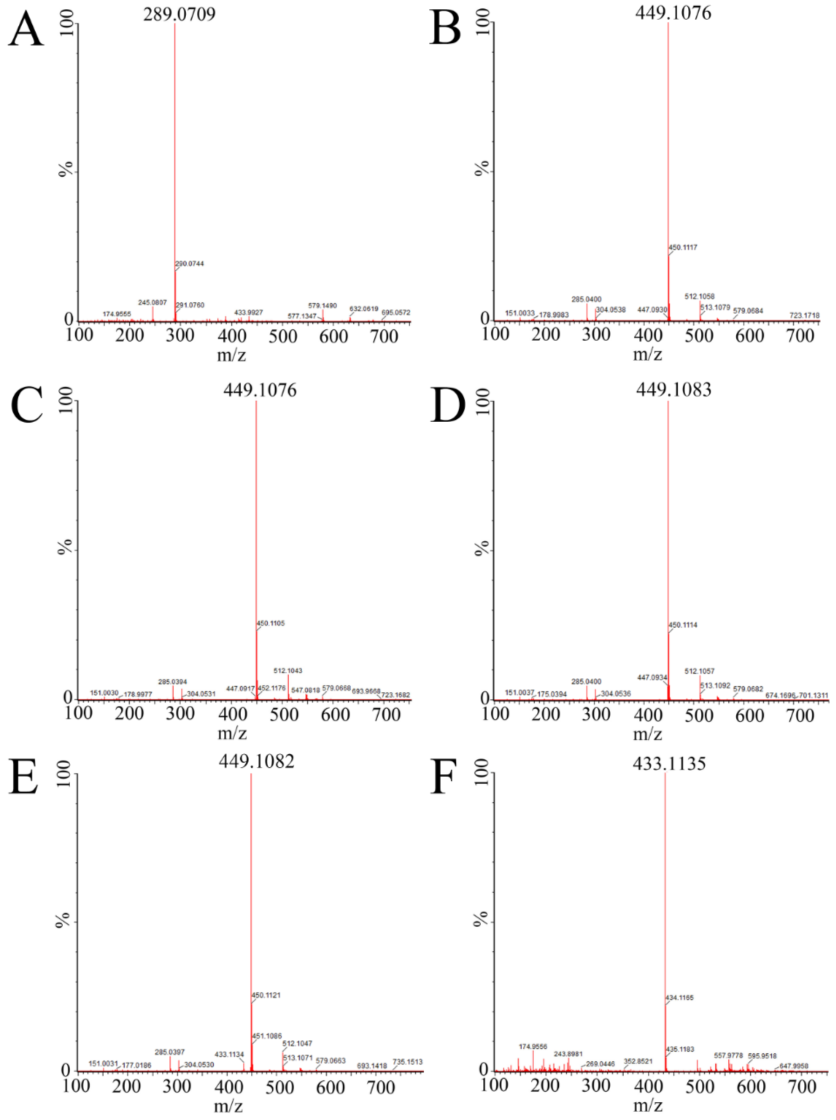

Figure S1. The mass spectra of six flavonoids in *Smilax glabra* flavonoids extract (SGF). A: (-)-epicatechin; B: neoastilbin; C: astilbin; D: neoisoastilbin; E: isoastilbin; F: engeletin.

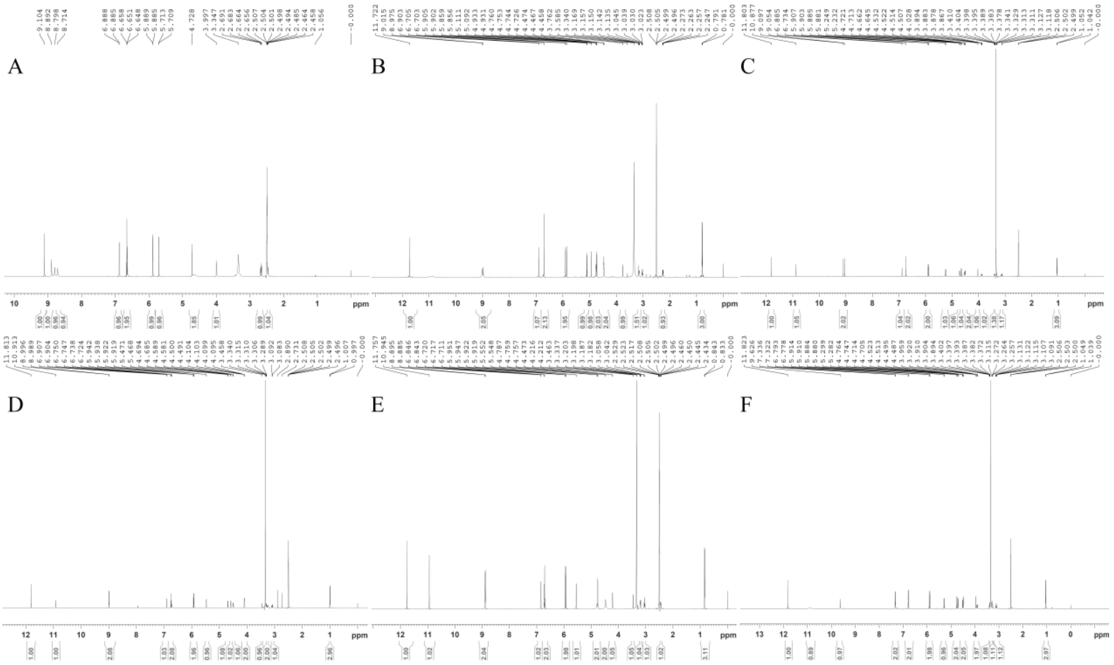

Figure S2. The ^{1}H NMR spectra of six flavonoids in *Smilax glabra* flavonoids extract (SGF). A: (-)-epicatechin; B: neoastilbin; C: astilbin; D: neoisoastilbin; E: isoastilbin; F: engeletin.

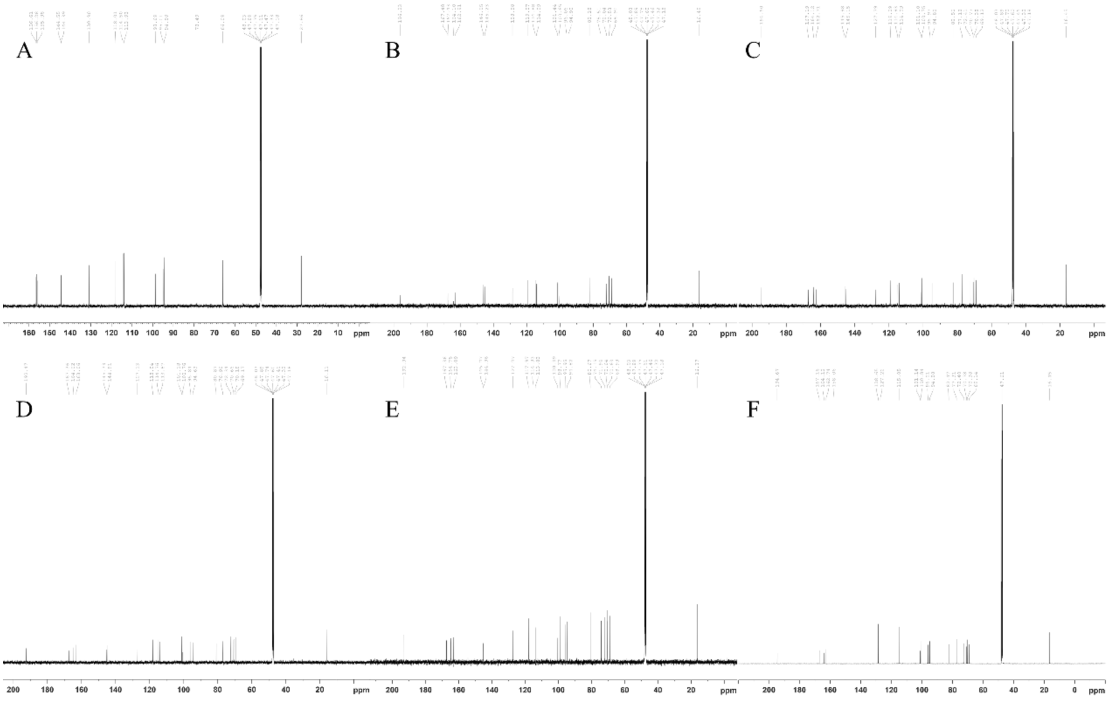

Figure S3. The ^{13}C NMR spectra of six flavonoids in *Smilax glabra* flavonoids extract (SGF). A: (-)-epicatechin; B: neoastilbin; C: astilbin; D: neoisoastilbin; E: isoastilbin; F: engeletin.

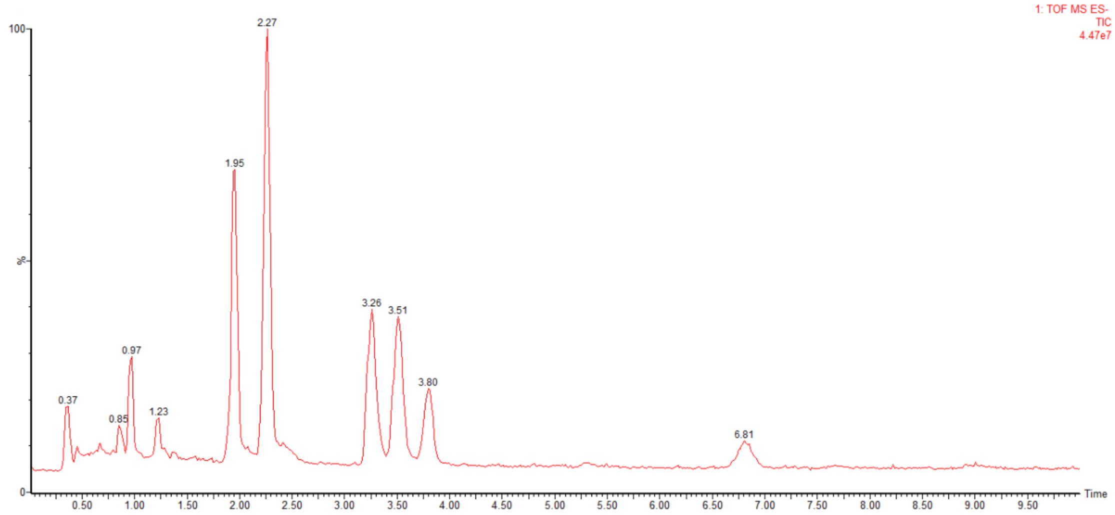

Figure S4. The total ion current (TIC) of *Smilax glabra* flavonoids extract (SGF).
